# Risk of Lymphoma and Solid Cancer among Patients with Rheumatoid Arthritis in a Primary Care Setting

**DOI:** 10.1371/journal.pone.0099388

**Published:** 2014-06-10

**Authors:** Christen Lykkegaard Andersen, Hanne Lindegaard, Hanne Vestergaard, Volkert Dirk Siersma, Hans Carl Hasselbalch, Niels de Fine Olivarius, Ole Weis Bjerrum, Peter Junker

**Affiliations:** 1 Department of Hematology, Roskilde University Hospital, Roskilde, Denmark; 2 The Research Unit for General Practice and Section of General Practice, Department of Public Health, University of Copenhagen, Copenhagen, Denmark; 3 Department of Rheumatology, Odense University Hospital, Odense, Denmark; 4 Department of Hematology, Odense University Hospital, Odense, Denmark; 5 Department of Hematology, Copenhagen University Hospital, Copenhagen, Denmark; Queen's University Belfast, United Kingdom

## Abstract

**Background:**

Several studies have demonstrated an association between rheumatoid arthritis (RA) and lymphoproliferative malignancies, but pathogenic mechanisms remain unclear. We investigated 1) the risk of lymphoproliferative malignancies and solid tumors in adults with RA identified in primary care and 2) the possible mediating role of blood eosinophilia in the clonal evolution of cancer in these patients.

**Methods:**

From the Copenhagen Primary Care Differential Count (*CopDiff*) Database, we identified 356,196 individuals with at least one differential cell count (DIFF) encompassing the eosinophil count between 2000–2007. From these, one DIFF was randomly chosen (the index DIFF). By linking to the Danish National Patient Register, we categorized the selected individuals according to known longstanding (≥3 years) or recent onset (<3 years) RA prior to the index DIFF. In addition, the cohort was stratified according to management in primary or secondary care. From the Danish Cancer Registry we ascertained malignancies within four years following the index DIFF. Using multivariable logistic regression, odds ratios (OR) were calculated and adjusted for sex, age, year, month, eosinophilia, comorbid conditions and C-reactive protein (CRP).

**Results:**

921 patients had recent onset RA and 2,578 had longer disease duration. Seventy three percent of RA patients were managed in primary care. After adjustment for sex, age, year, and month, neither recent onset nor long-standing RA was associated with incident lymphoproliferative malignancies or solid cancers. These risk estimates did not change when eosinophilia, CRP, and comorbidities were included in the models.

**Conclusions:**

In this large cohort of patients with RA of short or long duration recruited from a primary care resource, RA was not associated with an increased risk of lymphoproliferative or solid cancers during 4 years of follow-up, when the models were adjusted for confounders. Blood eosinophilia could not be identified as a mediator of cancer development in the present setting.

## Introduction

The association between rheumatoid arthritis (RA) and cancer has been investigated in several mainly hospital-based studies. These have indicated that the overall cancer occurrence in patients with RA is only marginally higher than in healthy individuals, while elevated risk figures have been reported for several specific cancers including leukemia [1], non-melanoma skin cancers [2], and lung cancer [3], [4]. In particular, an excess risk ranging from 50% to 200% of lymphoproliferative malignancies, especially diffuse large B-cell lymphoma, has been consistently reported in previous studies on RA and cancer [5]–[7]. The relative risks of cancer have been reported to be highest within the first year of follow-up although increased risk figures have been observed after up to 20 years of follow-up [8], [9].

Two major pathways have been suggested to account for the association between RA and malignancy: extrinsic pro-oncogenic effects of disease-modifying antirheumatic drugs (DMARDs) [10], [11]; and intrinsic pro-oncogenic effects related to disease activity [6]–[8], [12]–[14]. Recent studies indicate that RA patients with active disease are at increased risk of malignant lymphomas, compared with RA patients with low disease activity or remission [6]–[8], [12]–[14]. Thus, the pathways by which active RA impose an increased risk of lymphoma are poorly understood. In this context, the eosinophilic granulocyte is a pertinent candidate. Eosinophilia (≥0.5×10^9^ eosinophils/l peripheral blood) may be induced by infections and inflammation, including inflammatory processes accompanying solid and hematological malignancies [15]–[17]. Activation of eosinophils may lead to organ damage, irrespective of the cause of the eosinophilia [16], [18], [19]. It has been reported that eosinophilia is a rather common finding among patients in rheumatologic outpatient clinics, with an estimated prevalence of 7.7% [20]. Besides, eosinophilia has been linked to prognosis and severity of extra-articular manifestations in RA [21]. Taken together, these observations support the view that eosinophilia may be associated with cancer risk in RA.

The aim of the present study is twofold: 1) to investigate the risk of lymphoproliferative malignancies and solid tumors in adult RA identified in a primary care setting, and 2) to investigate the possible mediating role of blood eosinophilia in the clonal evolution of cancer in these patients.

## Methods

### Ethics Statement

The study was approved by The Danish Data Protection Agency (journal no: 2013-54-0507), and did not need approval by an institutional review board or ethical review board according to Danish legislation. Patient information was anonymized and de-identified prior to analysis and no clinical records were used. Patient consent is not mandatory for this type of study in Denmark.

### Patients

**Figure 1:**
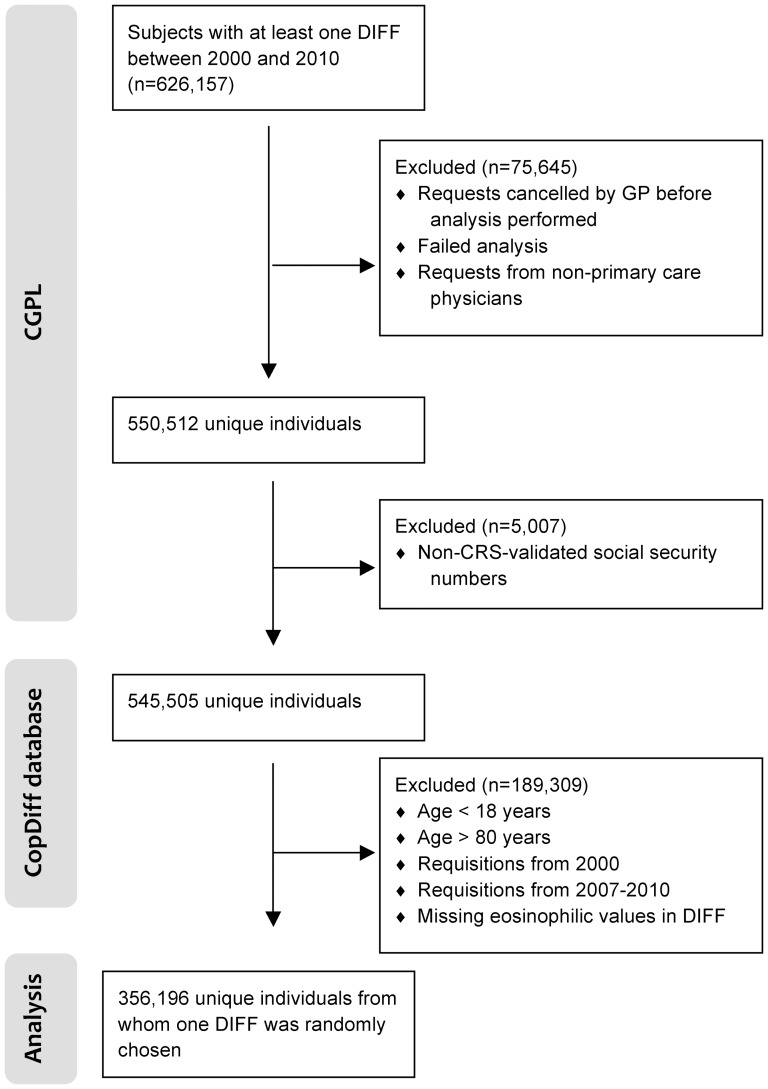
Flowchart for the study population. CGPL, Copenhagen General Practitioners' Laboratory; CopDiff, Copenhagen Primary Care Differential Count Database; CRS, The Danish Civil Registration System; DIFF, differential cell count; GP, general practitioner.

**Figure 2:**
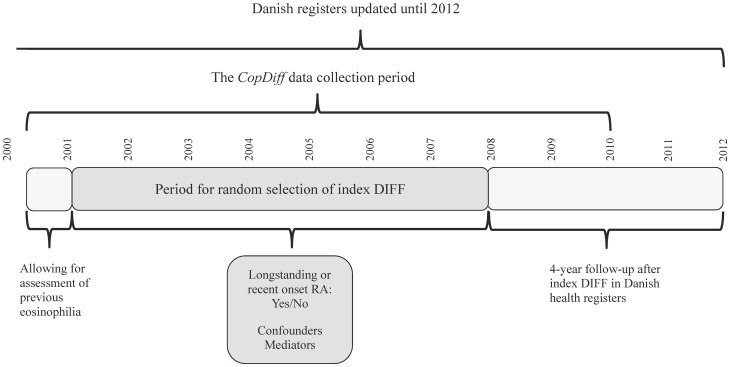
Timeline for index DIFF selection.

The Copenhagen General Practitioners' Laboratory (CGPL) (renamed the Elective Laboratory of the Capital Region from 01.01.2013) provides laboratory services to all general practitioners (GPs) in the Copenhagen area, covering approximately 1.2 million inhabitants. CGPL has International Organization for Standardization (ISO) accreditation and has stored all laboratory data since 01.05.2000. The Copenhagen Primary Care Differential Count (*CopDiff*) Database contains results from all differential cell counts (DIFF) requested by GPs in Copenhagen from 01.05.2000 to 25.01.2010. From each of the 359,950 unique, adult individuals (aged 18 to 80 years) with at least one DIFF in the period 01.01.2001 to 31.12.2007, one DIFF (i.e. the index DIFF) encompassing the eosinophil count was chosen by computer-generated random numbers (n = 356,196), see (Figure 1). The selected individuals were categorized according to prevalent blood eosinophilia (Figure 2). Where available, the level of C-reactive-protein (CRP), categorized as “increased” (≥10 mg/L) vs. “normal” (<10 mg/L), was also obtained (n = 229,511). Furthermore, we recorded whether another DIFF had been done within 6 months before the request (n = 32,475) and whether eosinophilia was also present in this DIFF. We found that our strategy of randomly choosing one DIFF per individual to assess incidences of the specified outcomes was feasible for two reasons. First, it meant that we did not to have to control for individuals that entered the cohort multiple times at different points in time. Second, we sought to minimize surveillance bias which seemed more likely to occur if, for example, we had opted for “the first DIFF” or “the DIFF closest to the outcome of interest” [22]. The period we chose for index DIFF selection was from 01.01.2001, rather than from the beginning of the *CopDiff* database (01.05.2000), because we wanted to assess whether a possible eosinophilia was longstanding (within 6 months before the index DIFF). Also, since all individuals were to have a fixed 4-year follow up after the index DIFF, we excluded DIFFs after 31.12.2007 (Figure 2).

In November 2013, the *CopDiff* database was linked to The Danish Cancer Registry (DCR), which contains data on all malignancies in Denmark since 1942 and to which reporting is mandatory [23]; and to The Danish National Patient Register (NPR), which contains information on all contacts with hospitals in Denmark with discharge diagnoses and surgical procedures [24]. The NPR also includes outpatient clinic contacts. To adjust for possible confounding by comorbid conditions, we computed Charlson's Comorbidity Index (CCI) [25] from hospital contacts recorded in the NPR for three years before the index DIFF. Information on occurrence of RA prior to the index DIFF was assessed from hospital contacts in the NPR and based on International Classification of Disease, ICD-8 codes: 712.39 (used from 1977–1993) and ICD-10 codes: M05, M06 (used from 1994-present). RA was categorized as recent (<3 years) or longstanding (≥3 years) according to date of first occurrence (since 1977) prior to the index DIFF. Previously, Pedersen *et al* reported that RA diagnoses in the NPR based on recordings from hospitals can be utilized for epidemiological purposes if inherent data limitations are considered [26]. Patients were defined as having “primary care managed RA” if they had been referred back to primary care at their most recent contact with a rheumatology department prior to the index DIFF.

Outcomes were defined as the incidences of the following diagnoses recorded in DCR over the four-year period following the index DIFF: 1) lymphoproliferative malignancies defined as ICD-10 codes: C81, C82–C85, and C91; and ICD for Oncology (ICD-O) morphological codes within the range: 9590/3/-9729/3/. Diffuse large B-cell lymphoma defined as ICD-10 code C83.3 and ICD-O morphological codes 96793, 96803, and 96843. Solid cancer defined as: buccal cavity and pharynx (C00-C14; C462), digestive organs (C15–C26), respiratory system including thoracic organs (C30–39; C450), bones, joints and articular cartilage (C40–C41), skin (C43–C44; C460), mesothelium and connective tissue (C451–C459; C461; C463; C467; C468; C469; C47–C49; B210), breast (C50), female genital organs (C51–C58), male genital organs (C60–C63), urinary tract (C64–C68; D090–D091; D301–D309; D411–D419), eye and central nervous system (C69–C72; C751–C753; D32–D33; D352–D354; D42–D43; D443–D445), and endocrine glands (C73–C74; C750; C754–C759).

### Statistical analysis

**Figure 3:**
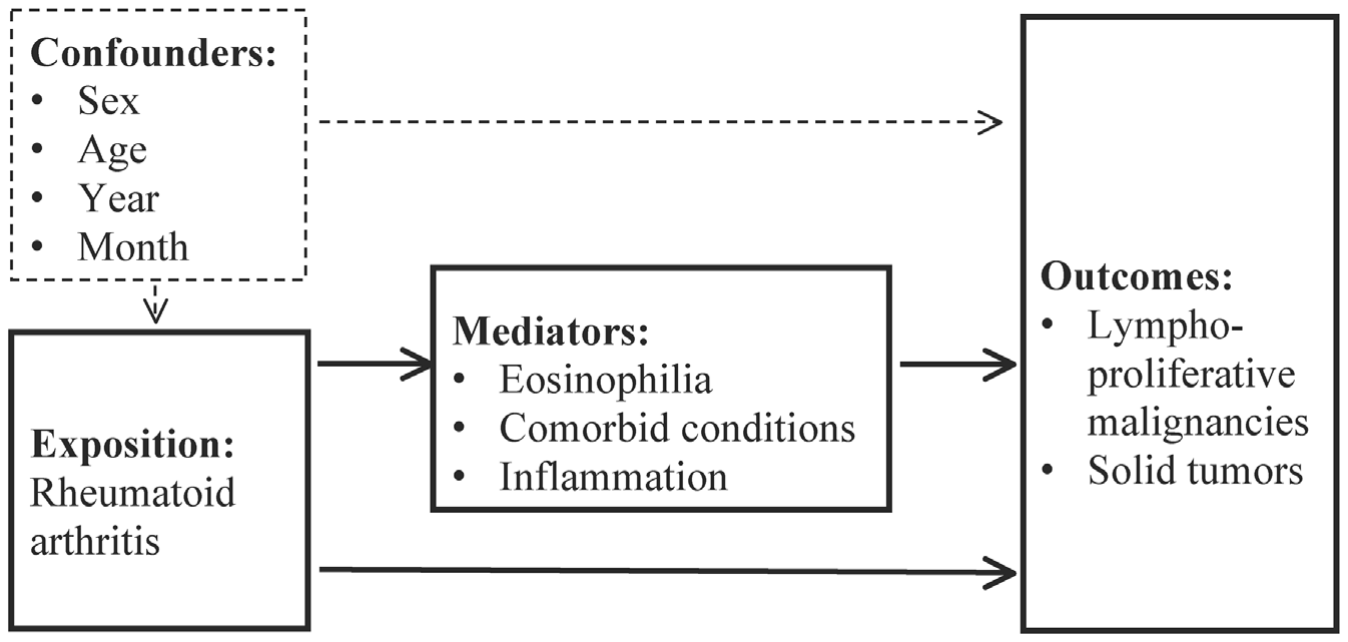
Flowchart illustrating the background for analytical approaches.

In order to pursue our first aim of investigating the risk of lymphoproliferative malignancies and solid tumors in adults with identified RA, we used multivariable logistic regression to compute odds ratios (ORs) with 95% confidence intervals (CIs) for the four-year incidence of lymphoproliferative malignancies and solid cancers following the index DIFF. The ORs were reported without any adjustment, and again after adjusting for potential confounders (sex, age [quadratic], year, and month of DIFF sampling). Regarding our second aim, to investigate the possible mediating role of blood eosinophilia in the clonal evolution of cancer in RA patients, we used the same model with potential confounders, but adjusted for candidate mediators of disease (eosinophilia, competing comorbid conditions, and inflammation) in a stepwise fashion in order to analyze the impact of the mediators sequentially. An overview of the variables included in the analyses is seen in Figure 3. Individuals who had already been diagnosed with a cancer (recorded in the DCR since 1977) were excluded from risk analyses. To account for multiple statistical testing, P-values less than 0.0033 were regarded as significant, as this controls the false discovery rate at 5% using the Benjamini-Hochberg method [27]. All analyses and calculations were performed with SAS version 9.2 (SAS Institute Inc., Cary, NC, USA).

## Results

The mean age (SD) of the entire study population was 48.3 years (16.7) with a female:male ratio of 1.38 (208,691/151,259); see (Table 1). Since 1977 and before the index DIFF, 1,167 patients (0.3%) had been diagnosed with a lymphoproliferative cancer and 22,541 (6.3%) had experienced a solid cancer. A total of 14.406 patients (4.0%) had eosinophilia.

**Table 1:** Baseline subject characteristics.

	Rheumatoid arthritis			
	No, *n = 356*,*451*	Longstanding (≥3 years), *n = 2,578*	Recent onset (<3 years), *n = 921*	Total, *n = 359,950*
Gender				
Male	150,385 (42,2)	652 (25.3)	222 (24.1)	151,259 (42.0)
Female	206,066 (57.8)	1,926 (74.7)	699 (75.9)	208,691 (58.0)
Age, years	48.2±16.7	60.2±14.0	56.9±14.5	48.3±16.7
Eosinophilia				
No (<0.5•10^9^/L)	338,502 (96.0)	2,414 (94.8)	874 (95.3)	341,790 (96.0)
Yes (≥0.5•10^9^/L)	14,231 (4.0)	132 (5.2)	43 (4.7)	14,406 (4.0)
Previous lymphoproliferative cancer [1]				
No	355,301 (99.7)	2,565 (99.5)	917 (99.6)	358,783 (99.7)
Yes	1,150 (0.3)	13 (0.5)	4 (0.4)	1,167 (0.3)
Previous solid tumor				
No	334,285 (93.8)	2,295 (89.0)	829 (90.0)	337,409 (93.7)
Yes	22,166 (6.2)	283 (10.9)	92 (10.0)	22,541 (6.3)
Charlson's Comorbidity Index [2]	0.21±0.76	0.82±1.22	1.56±1.23	0.22±0.77
C-reactive protein				
No blood test	128,624 (36.2)	836 (32.5)	295 (32.1)	129,755 (36.1)
Normal	179,739 (50.5)	1,019 (39.6)	404 (44.0)	181,162 (50.4)
Increased [3]	47,413 (13.3)	717 (27.8)	219 (23.9)	48,349 (13.5)
Previous eosinophilia (<6 months)				
No blood test	324,665 (91.1)	2,112 (81.8)	698 (75.8)	327,475 (91.0)
Only negative tests	29,465 (8.3)	432 (16.7)	207 (22.5)	30,104 (8.3)
At least one positive test [4]	2,316 (0.6)	39 (1.5)	16 (1.7)	2,371 (0.7)
Incident lymphoproliferative cancer (≤4 years) [1]				
No	354,432 (99.8)	2,553 (99.5)	915 (99.8)	357,900 (99.8)
Yes	869 (0.2)	12 (0.5)	2 (0.2)	883 (0.2)
Incident solid tumor (≤4 years)				
No	319,003 (95.4)	2,102 (91.6)	781 (94.2)	321,886 (95.4)
Yes	15,282 (4.6)	193 (8.4)	48 (5.8)	15,523 (4.6)

Values are number (column-%) or means (SD). [1] Lymphoproliferative cancer defined as Hodgkin's lymphoma, non-Hodgkin lymphoma and chronic lymphocytic leukemia, [2] Calculated on previous hospital contacts (<3 years), [3]>10 mg/L, [4]>0.5•10^9^/L.

Of 359,950 individuals, 3,499 (1.0%) had been diagnosed with RA according to the NPR. Of these, 921 were diagnosed during the three years prior to the index DIFF (recent onset), and 2,578 were diagnosed earlier than this (long-standing RA); see (Table 1). The mean age of patients with recent onset and longstanding RA was 56.9 and 60.2 years respectively, with a female:male ratio of 3.15 and 2.95. Seventy-three percent of RA patients were managed in primary care at the time of the index DIFF blood sampling. In the subsequent four-year period, fourteen RA patients developed lymphoproliferative cancer and a solid cancer was diagnosed in 241 RA patients (Table 1). The overall incidences of lymphoproliferative cancer and solid cancers over the four-year period following the index count were 61 and 1,078 per 100,000 person-years, respectively. Additional baseline subject characteristics are presented in Table 1.

In unadjusted analyses, patients with longstanding RA had an increased risk for lymphoproliferative cancer (OR [95% CI] = 1.92 [1.08–3.39]) and solid cancer (1.92 [1.65–2.22]), while the corresponding risk estimates were close to unity among patients with recent onset RA (Table 2). When adjusting for demographic characteristics, neutral ORs for lymphoproliferative or solid cancer were also found for patients with longstanding RA (1.31 [0.74–2.33] and 1.10 [0.94–1.28], respectively). Adjustment for eosinophilia, CRP and comorbidities did not alter any of the risk estimates. When performing subgroup analysis for diffuse large B-cell lymphoma, and including confounders and mediators in the models, we did not observe any association with RA (ORs of 1.41 [0.44–4.45] and 1.88 [0.25–13.96] for longstanding and recent onset RA, respectively). These estimates did not change when excluding confounders and mediators: ORs were 2.17 (0.69-6.81) and 2.03 (0.28–14.49) for longstanding and recent onset RA, respectively.

**Table 2:** The association between longstanding or recent onset rheumatoid arthritis and 4-year incidence of cancer.

Rheumatoid arthritis	Adjustment	4-year incidence of lymphoproliferative cancer *		4-year incidence of solid cancer	
No	None	1.00	0.13^†^	1.00	<0.0001^†^
Longstanding (≥3 years)		1.92 (1.08–3.39)	0.026	1.92 (1.65–2.22)	<0.0001
Recent onset (<3 years)		0.89 (0.22–3.58)	0.87	1.28 (0.96–1.72)	0.094
No	[1]	1.00	0.59^†^	1.00	0.33^†^
Longstanding (≥3 years)		1.31 (0.74–2.33)	0.35	1.10 (0.94–1.28)	0.23
Recent onset (<3 years)		0.72 (0.18–2.88)	0.64	0.88 (0.65–1.18)	0.38
No	[1], [2]	1.00	0.64^†^	1.00	0.26^†^
Longstanding (≥3 years)		1.29 (0.71–2.34)	0.41	1.10 (0.95–1.28)	0.21
Recent onset (<3 years)		0.72 (0.18–2.88)	0.64	0.86 (0.63–1.15)	0.31
No	[1], [2], [3]	1.00	0.51^†^	1.00	0.27^†^
Longstanding (≥3 years)		1.46 (0.80–2.66)	0.22	1.11 (0.96–1.30)	0.17
Recent onset (<3 years)		1.01 (0.25–4.09)	0.99	0.88 (0.65–1.19)	0.40
No	[1], [2], [3], [4]	1.00	0.66^†^	1.00	0.42^†^
Longstanding (≥3 years)		1.34 (0.73–2.44)	0.35	1.06 (0.91–1.23)	0.46
Recent onset (<3 years)		0.95 (0.23–3.88)	0.95	0.85 (0.63–1.15)	0.29

Values are odds ratios and *p*-values from multivariable logistic regression analyses for the development of lymphoproliferative cancer and solid tumors for the risk groups: No previous rheumatoid arthritis, Longstanding rheumatoid arthritis, and Recent onset rheumatoid arthritis. Odds ratios are adjusted in a stepwise manner: [1] sex, age (quadratic), year and month; [2] previous and present eosinophilia; [3] Charlson's Comorbidity Index, and [4] C-reactive protein. ^†^ *P*-value for the likelihood-ratio test of all categories of eosinophilia simultaneously. * Hodgkin's lymphoma, non-Hodgkin lymphoma and chronic lymphocytic leukemia.

## Discussion

In this study based on patients in the *CopDiff* database, RA of recent or longstanding duration was not associated with an increased risk of lymphoproliferative malignancies or solid cancer within a 4-year time frame. Adjusting for the anticipated mediators, e.g. eosinophilia, inflammation, and comorbidities yielded similar results. While absence of an association with most solid cancers accords with previous results, the majority of earlier studies reported an excess risk of lymphoproliferative malignancies among RA patients [7]–[9], [12], [28]–[30]. However, the patients included in these studies were recruited on the basis of hospital contacts [4], [8], [30] or from RA registers [9], [12], [28], [29]. Data from RA registers in particular offer detailed patient data of high quality concerning RA, its course, complications, and comorbidities. Thus, studies based on either of these resources are in danger of reporting associations between RA and malignancies due to further examinations derived from increased awareness of the condition [22]. The *CopDiff* population was sampled continuously without any restrictions as to why the DIFF was requested by the GP which, together with the use of computer-generated random selection of index DIFFs, diminishes surveillance bias. Furthermore, the majority of RA patients in this study were managed in primary care. In Denmark, biological and complex non-biological therapies are offered exclusively in secondary care. Therefore, the majority of patients in the present cohort can be assumed to have mild disease.

The study has limitations. First, the *CopDiff* database is derived from a population which may present with elevated white blood cell counts and more background morbidity than the general population. The use of logistic regression analysis on the 4-year incidence, however, ensures that the measures of excess risk (OR) can be interpreted independently of the frequency of the cancers in the study, and the OR can therefore be considered to be a valid estimate of excess risk in the general population as well [31]. Second, the follow-up period of only 4 years was chosen in order to optimize the ability to link RA with incident cancer. Thus, we cannot exclude the view that some RA-related malignancies appeared beyond this time frame due to the latency period for cancer development. Third, information about drug treatment was not available. To our knowledge, only one study has found an association between the use of a specific group of antirheumatic drugs, the TNF-inhibitors, and cancer [10], while all other studies have failed to confirm these results [32], [33]. Finally, we did not have access to information on body mass index, smoking, alcohol consumption, exercise patterns, or family history of disease. Smoking is a well-recognized risk factor for the development of RA and lung cancer, and the prevalence of smoking among people with RA is relatively high [3]. However, these risk factors have not been clearly linked to lymphoproliferative malignancies [34], [35].

Particular strengths of this study include the fact that all malignant diagnoses were derived from the DCR which was established in 1942 and to which reporting is mandatory. The validity of the DCR is optimized through regular quality control routines applied in daily data production with manual coding and data validation. An incidence of solid cancers of 1.078 per 100.000 person-years also compares with the national incidence of 1.205 per 100.000 person-years [36]. This suggests that the *CopDiff* database participants do not differ significantly from the general population with respect to cancer incidence. In addition, we observed age and gender distributions among RA patients which compare well with figures reported from Nordic countries, thereby adding to the validity of the study [37], [38].

In conclusion, based on this large population of individuals identified in a primary care setting, neither early nor longstanding RA was associated with an increased risk of lymphoproliferative or solid cancers within a 4-year time frame. Patients with RA managed in the Danish primary health care system, generally have mild disease which might explain the absence of an association with lymphoproliferative malignancies reported previously among RA patients in secondary care. Blood eosinophilia could not be identified as a mediator of cancer development in the present setting.

## References

[1] Hemminki K, Liu X, Forsti A, Ji J, Sundquist J, et al (2013) Subsequent leukaemia in autoimmune disease patients. Br J Haematol 161: 677–687. 23565673 10.1111/bjh.12330

[2] Chakravarty EF, Michaud K, Wolfe F (2005) Skin cancer, rheumatoid arthritis, and tumor necrosis factor inhibitors. J Rheumatol 32: 2130–2135. 16265690

[3] Khurana R, Wolf R, Berney S, Caldito G, Hayat S, et al (2008) Risk of development of lung cancer is increased in patients with rheumatoid arthritis: a large case control study in US veterans. J Rheumatol 35: 1704–1708. 18634160

[4] Mercer LK, Davies R, Galloway JB, Low A, Lunt M, et al (2013) Risk of cancer in patients receiving non-biologic disease-modifying therapy for rheumatoid arthritis compared with the UK general population. Rheumatology (Oxford) 52: 91–98. 23238979 10.1093/rheumatology/kes350 PMC3521445

[5] Askling J (2007) Malignancy and rheumatoid arthritis. Curr Rheumatol Rep 9: 421–426. 17915099 10.1007/s11926-007-0067-1

[6] Baecklund E, Iliadou A, Askling J, Ekbom A, Backlin C, et al (2006) Association of chronic inflammation, not its treatment, with increased lymphoma risk in rheumatoid arthritis. Arthritis Rheum 54: 692–701. 16508929 10.1002/art.21675

[7] Baecklund E, Smedby KE, Sutton LA, Askling J, Rosenquist R (2014) Lymphoma development in patients with autoimmune and inflammatory disorders—what are the driving forces? Sem Cancer Biol 24: 61–70. 10.1016/j.semcancer.2013.12.001 24333759

[8] Ekstrom K, Hjalgrim H, Brandt L, Baecklund E, Klareskog L, et al (2003) Risk of malignant lymphomas in patients with rheumatoid arthritis and in their first-degree relatives. Arthritis Rheum 48: 963–970. 12687538 10.1002/art.10939

[9] Chen YJ, Chang YT, Wang CB, Wu CY (2011) The risk of cancer in patients with rheumatoid arthritis: a nationwide cohort study in Taiwan. Arthritis Rheum 63: 352–358. 21279991 10.1002/art.30134

[10] Bongartz T, Sutton AJ, Sweeting MJ, Buchan I, Matteson EL, et al (2006) Anti-TNF antibody therapy in rheumatoid arthritis and the risk of serious infections and malignancies: systematic review and meta-analysis of rare harmful effects in randomized controlled trials. JAMA 295: 2275–2285. 16705109 10.1001/jama.295.19.2275

[11] Cush JJ, Dao KH (2012) Malignancy risks with biologic therapies. Rheum Dis Clin North Am 38: 761–770. 23137581 10.1016/j.rdc.2012.09.006

[12] Franklin J, Lunt M, Bunn D, Symmons D, Silman A (2006) Incidence of lymphoma in a large primary care derived cohort of cases of inflammatory polyarthritis. Ann Rheum Dis 65: 617–622. 16249224 10.1136/ard.2005.044784 PMC1798140

[13] Smedby KE, Baecklund E, Askling J (2006) Malignant lymphomas in autoimmunity and inflammation: a review of risks, risk factors, and lymphoma characteristics. Cancer Epidemiol Biomarkers Prevention 15: 2069–2077. 10.1158/1055-9965.EPI-06-0300 17119030

[14] Wolfe F, Michaud K (2007) The effect of methotrexate and anti-tumor necrosis factor therapy on the risk of lymphoma in rheumatoid arthritis in 19,562 patients during 89,710 person-years of observation. Arthritis Rheum 56: 1433–1439. 17469100 10.1002/art.22579

[15] Andersen CL, Siersma VD, Hasselbalch HC, Lindegaard H, Vestergaard H, et al (2013) Eosinophilia in routine blood samples and the subsequent risk of hematological malignancies and death. American journal of hematology 88: 843–847. 23765950 10.1002/ajh.23515

[16] Gotlib J (2014) World Health Organization-defined eosinophilic disorders: 2014 update on diagnosis, risk stratification, and management. Am J Hematol 89: 325–337. 24577808 10.1002/ajh.23664

[17] Tefferi A, Patnaik MM, Pardanani A (2006) Eosinophilia: secondary, clonal and idiopathic. Br J Haematol 133: 468–492. 16681635 10.1111/j.1365-2141.2006.06038.x

[18] Cools J (2005) The hypereosinophilic syndrome: idiopathic or not, that is the question. Haematologica 90: 582–584. 15921372

[19] Jacobsen EA, Helmers RA, Lee JJ, Lee NA (2012) The expanding role(s) of eosinophils in health and disease. Blood 120: 3882–3890. 22936660 10.1182/blood-2012-06-330845 PMC3496950

[20] Kargili A, Bavbek N, Kaya A, Kosar A, Karaaslan Y (2004) Eosinophilia in rheumatologic diseases: a prospective study of 1000 cases. Rheumatol Int 24: 321–324. 15067429 10.1007/s00296-004-0469-6

[21] Panush RS, Franco AE, Schur PH (1971) Rheumatoid arthritis associated with eosinophilia. Ann Intern Med 75: 199–205. 4934278 10.7326/0003-4819-75-2-199

[22] Haut ER, Pronovost PJ (2011) Surveillance bias in outcomes reporting. JAMA 305: 2462–2463. 21673300 10.1001/jama.2011.822

[23] Gjerstorff ML (2011) The Danish Cancer Registry. Scand J Public Health 39: 42–45. 21775350 10.1177/1403494810393562

[24] Lynge E, Sandegaard JL, Rebolj M (2011) The Danish National Patient Register. Scand J Public Health 39: 30–33. 21775347 10.1177/1403494811401482

[25] Charlson ME, Pompei P, Ales KL, MacKenzie CR (1987) A new method of classifying prognostic comorbidity in longitudinal studies: development and validation. J Chron Dis 40: 373–383. 3558716 10.1016/0021-9681(87)90171-8

[26] Pedersen M, Klarlund M, Jacobsen S, Svendsen AJ, Frisch M (2004) Validity of rheumatoid arthritis diagnoses in the Danish National Patient Registry. Eur J Epidemiol 19: 1097–1103. 15678789 10.1007/s10654-004-1025-0

[27] Benjamini Y, Hochberg Y (1995) Controlling the False Discovery Rate: a Practical and Powerful Approach to Multiple Testing. J Roy Stat Soc B 57: 289–300.

[28] Abasolo L, Judez E, Descalzo MA, Gonzalez-Alvaro I, Jover JA, et al (2008) Cancer in rheumatoid arthritis: occurrence, mortality, and associated factors in a South European population. Semin Arthritis Rheum 37: 388–397. 17977580 10.1016/j.semarthrit.2007.08.006

[29] Geborek P, Bladstrom A, Turesson C, Gulfe A, Petersson IF, et al (2005) Tumour necrosis factor blockers do not increase overall tumour risk in patients with rheumatoid arthritis, but may be associated with an increased risk of lymphomas. Ann Rheum Dis 64: 699–703. 15695534 10.1136/ard.2004.030528 PMC1755491

[30] Mellemkjaer L, Linet MS, Gridley G, Frisch M, Moller H, et al (1996) Rheumatoid arthritis and cancer risk. Eur J Cancer 32A: 1753–1757. 8983286 10.1016/0959-8049(96)00210-9

[31] Woodward M (2005) Epidemiology: Study Design And Data Analysis. Chapman & Hall/CRC: 278–280.

[32] Lopez-Olivo MA, Tayar JH, Martinez-Lopez JA, Pollono EN, Cueto JP, et al (2012) Risk of malignancies in patients with rheumatoid arthritis treated with biologic therapy: a meta-analysis. JAMA 308: 898–908. 22948700 10.1001/2012.jama.10857

[33] Ramiro S, Gaujoux-Viala C, Nam JL, Smolen JS, Buch M, et al (2014) Safety of synthetic and biological DMARDs: a systematic literature review informing the 2013 update of the EULAR recommendations for management of rheumatoid arthritis. Ann Rheum Dis 73: 529–535. 24401994 10.1136/annrheumdis-2013-204575

[34] Lichtman MA (2010) Obesity and the risk for a hematological malignancy: leukemia, lymphoma, or myeloma. Oncologist 15: 1083–1101. 20930095 10.1634/theoncologist.2010-0206 PMC3227901

[35] Morton LM, Hartge P, Holford TR, Holly EA, Chiu BC, et al (2005) Cigarette smoking and risk of non-Hodgkin lymphoma: a pooled analysis from the International Lymphoma Epidemiology Consortium (interlymph). Cancer Epidemiol Biomarkers Prev 14: 925–933. 15824165 10.1158/1055-9965.EPI-04-0693

[36] The Danish Cancer Registry 2010. http://www.sst.dk/publ/Publ2011/DAF/Cancer/Cancerregisteret2010.pdf

[37] Simonsson M, Bergman S, Jacobsson LT, Petersson IF, Svensson B (1999) The prevalence of rheumatoid arthritis in Sweden. Scand J Rheumatol 28: 340–343. 10665738 10.1080/03009749950155319

[38] The nationwide Danish DANBIO registry. https://danbio-online.dk/Danbio-yearly-report-2007-eng.pdf/view

